# Performance Evaluation of the Quantamatrix QMAC-dRAST System for Rapid Antibiotic Susceptibility Testing Directly from Blood Cultures

**DOI:** 10.3390/microorganisms10061212

**Published:** 2022-06-14

**Authors:** Manon Rosselin, Guy Prod’hom, Gilbert Greub, Antony Croxatto

**Affiliations:** 1 Institute of Microbiology, Lausanne University Hospital and University of Lausanne, 1011 Lausanne, Switzerland; manon.rosselin@unilabs.com (M.R.); guy.prodhom@chuv.ch (G.P.); gilbert.greub@chuv.ch (G.G.); 2 Unilabs, 1296 Coppet, Switzerland; 3 ADMED Microbiology, 2300 La Chaux-de-Fonds, Switzerland

**Keywords:** rapid, antibiotic, AST, blood, Quantamatrix, QMAC-dRAST

## Abstract

Objectives: Rapid antibiotic susceptibility testing (AST) for positive blood cultures can improve patient clinical outcomes if the time to an effective antimicrobial therapy is shortened. In this study, we tested the Quantamatrix dRAST system (QMAC-dRAST), a rapid AST system based on time-lapse microscopic imagery of bacterial colony formation in agarose. Methods: Evaluation of the QMAC-dRAST was performed from 250 monobacterial blood cultures including 130 Enterobacterales, 20 non-fermentative Gram-negative bacteria, 69 staphylococci and 31 enterococci. Blood cultures were recovered from anonymous patients or from spiking experiments to enrich our study with bacterial species and resistant strains. Categorical agreement (CA), minor errors (me), major errors (ME) and very major errors (VME) were calculated in comparison to the results obtained from the BD Phoenix™ M50. Discrepancies between the Phoenix™ M50 and QMAC-dRAST results were investigated using the gradient strip method. The repeatability and reproducibility performance of the QMAC-dRAST was assessed for 16 strains, each strain being tested five times from a spiked blood culture. Results: The overall CAs for Enterobacterales, non-fermentative Gram-negative bacteria, staphylococci and enterococci were 95.1%, 91.2%, 93.4% and 94.5%, respectively. The VME percentage was below 4% for all the groups except for staphylococci, which showed a VME rate of 7%. The median time to result was 6.7 h (range: 4.7–7.9). Repeatability and reproducibility assays showed a high reliability of AST results with best and worst ratios of 98.8% and 99.6% and 95.0% and 98.3%, respectively. Conclusions: The QMAC-dRAST is a fast and reliable system to determine AST directly from monobacterial blood cultures with a major TAT reduction compared to conventional AST testing.

## 1. Introduction

Over 1,200,000 persons develop bloodstream infections each year in Europe with a mortality rate of 21.6–37.8 per 100,000 person-years [1]. Rapid administration of an effective targeted antibiotherapy improves patient clinical outcomes by decreasing mortality and morbidity [1,2,3]. It is also associated with a shorter stay in hospital, thereby reducing the costs of care [4]. Conventional AST procedures usually consist of subculturing positive blood samples overnight to measure AST the following day directly from isolated bacterial colonies. Under these conditions antibiotic susceptibility profiles are typically delivered 24 to 72 h after blood culture positivity [5,6]. 

Reducing the time to when the AST results of a bloodstream infection are available is challenging, and different techniques and automates have been developed for this purpose [7,8]. Innovative technologies are currently being widely assessed in routine laboratories such as the detection of antimicrobial resistance by single-cell morphology analysis or nano-mechanical sensors [9,10]. However, most of these methods are currently not suitable for routine AST and new technologies are still needed for faster diagnosis of bloodstream infection. 

The Quantamatrix dRAST (QMAC-dRAST) device is a rapid and automated system, which allows AST directly from blood. It is composed of a microfluidic agarose channel (MAC) system that immobilizes bacteria in agarose-containing microfluidic chambers. Bacterial growth under different antibiotic culture conditions is tracked by time-lapse imaging [11]. In this study, accuracy and repeatability of the QMAC-dRAST were evaluated in comparison to the Becton Dickinson (BD) Phoenix™ M50 device, a well-validated automated AST system [12,13,14,15,16].

## 2. Methods

**Samples**. Evaluation of the QMAC-dRAST v2.5 in comparison to the Phoenix™ M50 (BD, Franklin Lakes, NJ, USA) was conducted from 250 blood cultures in the bacteriology laboratory of the Lausanne University hospital, Switzerland. Monobacterial blood cultures were recovered from anonymous patients or from spiking experiments. All blood culture samples were incubated on the BD BACTEC™ FX system (BD, USA). Gram staining was performed to determine whether a Gram-positive or a Gram-negative antibiotic panel was required and to exclude mixed blood cultures.

**Spiking experiments**. All the bacteria were thawed and subcultured twice onto Columbia agar plates at 37 °C in 5% CO_2_ atmosphere. Bacterial identification was confirmed by MALDI-TOF on a Microflex LT instrument (Bruker Daltonics). Bacterial suspensions diluted in 0.85% sodium chloride buffer were adjusted to defined bacterial concentrations: 1.5 × 10^1^ bacteria/mL for *Pseudomonas aeruginosa*, staphylococci and enterococci, and 0.3 × 10^1^ bacteria/mL for Enterobacterales. These concentrations were chosen to reflect the physiological conditions of bloodstream infections. BD BACTEC™ Plus Aerobic or Anaerobic media were inoculated with 5 mL of these suspensions and incubated in the BD BACTEC™ FX system until flagged positive. The median time of positivity for spiked blood cultures was 11.5, 14.1, 15.5 and 11.5 h for Enterobacterales, non-fermentative Gram-negative bacilli (GNB), *Staphylococcus* and *Enterococcus* bacteria, respectively.

**Antibiotic susceptibility testing**. Positive blood cultures were collected and bacterial pellets were prepared with the Rapid BACpro^®^ II kit (Nittobo Medical Co., Ltd., Tokyo, Japan) [17] for bacterial identification by MALDI-TOF on a Microflex LT instrument (Bruker Daltonics) and for Gram staining. Gram staining was performed from both native positive blood culture and from bacterial pellets to characterize Gram-positive and Gram-negative bacteria when no MALDI-TOF identification was obtained and to exclude mixed blood cultures. A 500 µL blood sample was introduced in the QMAC-dRAST system using either a Gram-positive or a Gram-negative panel. For the Phoenix™ M50 (BD) standard methods, blood samples were subcultured for 18 h to 24 h at 37 °C in 5% CO_2_ on Columbia agar plates (BD). Bacterial colonies were identified by conventional MALDI-TOF and 25 µL of a 0.5 MacFarland bacterial suspension was prepared from isolated colonies to perform a NMIC-502 g-negative panel or in a PMIC-96 g-positive panel. Tetracycline, piperacillin and erythromycin were not included for the comparison between the QMAC-dRAST and the Phoenix™ M50 since these antibiotics are not recommended as first-line therapy for bloodstream infections. Rifampicin was also excluded from our evaluation as the minimal MIC calculated by the Phoenix™ M50 did not discriminate between susceptible (S) and susceptible at increased exposure (I) interpretations according to the 2019 EUCAST guidelines. Colistin was not analyzed since the only recommended method is broth microdilution.

**Characterization of resistant phenotypes.** Resistant phenotypes were characterized according to AST results combined with standard methods of detection. For extended spectrum β-lactamases (ESBL), synergy tests between diffusion disks of clavulanate and cephalosporins were used as well as PM-PML gradient strips (bioMérieux, Marcy-l’Étoile, France), NG-Test^®^ CTX-M Multi (NG Biotech, Guipry, France) or BETA-LACTA™ chromogenic hydrolysis-based assays [18]. Carbapenemase producers were detected with NG-Test^®^ CARBA 5 (NG Biotech) or Carba NP tests [19]. Twelve carbapenemases were tested including seven OXA-48, one OXA-23, one IMP, one KPC-2 and two uncharacterized carbapenemases. Fifty-one Enterobacterales producing an AmpC β-lactamases (acquired or chromosomal) were tested with CN-CNI gradient strips (Biomerieux). Three natural and eight acquired vancomycin-resistant enterococci (VRE) were included in the study, the latter being identified using selective media (Oxoid) and the Xpert vanA/vanB molecular assays (Cepheid). Twenty-nine methicillin-resistant staphylococci were characterized with cefoxitin screens and Xpert MRSA/SA BC assays (Cepheid, Sunnyvale, CA, USA).

**Repeatability and reproducibility**. Analysis of AST measurement repeatability and reproducibility was performed on 8 GNB and 8 Gram-positive cocci (GPC), each strain being tested 5 times from a single blood culture for repeatability and from 5 different blood cultures for reproducibility. The mode values correspond to the most frequently occurring minimal inhibitory concentration (MIC) result for each antibiotic tested by the QMAC-dRAST. The best and worst cases were calculated, the best case calculation assuming the off-scale result is within one well from the mode and the worst case calculation assuming the off-scale result is greater than one well from the mode [20].

**Evaluation of the QMAC-dRAST performance**. Categorical agreement (CA), minor errors (me), major errors (ME) and very major errors (VME) were defined according to the US Food and Drug Administration (FDA) [20]. CA is defined as agreement of test results interpreted within the same susceptibility category (S/I/resistant (R)). AST discordance results were classified as VME (reported S with the QMAC-dRAST when R with the Phoenix™ M50), ME (reported R when S with the Phoenix™ M50) and me (reported I when S or R on the Phoenix™ M50 or reported R or S when I on the Phoenix™ M50). The percentage of ME and VME were evaluated with the total number of strains susceptible or resistant to each antibiotic, respectively. Discrepancies between QMAC-dRAST and Phoenix™ M50 results (ME with a difference in MIC values > 2 two-fold dilutions and all the VME) were investigated using MIC gradient strips on Mueller-Hinton agar (Oxoid) following EUCAST guidelines. Differences in results for clindamycin-inducible resistance were resolved via a D-test (Oxoid) [21]. For oxacillin discrepancies, a cefoxitin disk (Oxoid) was used. Isolates were classified as S/I/R following the 2019 EUCAST breakpoints. Quality controls for the QMAC-dRAST and the BD Phoenix™ M50 were performed according to the manufacturer’s recommendations using the indicated ATCC isolates.

## 3. Results

### 3.1. QMAC-dRAST Performance

To assess the QMAC-dRAST performance, 250 bacterial strains were evaluated from 56 blood cultures from anonymous patients and 194 spiked blood cultures. Spiked blood cultures were included to enrich our study in bacterial species and in antimicrobial resistance mechanisms (Table 1). Overall, 130 Enterobacterales, 20 non-fermentative Gram-negative bacilli (GNB), 69 staphylococci and 31 enterococci were evaluated (Table 1). The antibiotics tested for each bacterial group are shown in Supplementary Table S1. Essential agreement was not calculated as most of the reference and QMAC-dRAST MIC results fell in the less than or greater than categories, which are not evaluable according to the FDA guidelines (Supplementary Tables S2–S25) [20].

The rates of CA, VME, ME and me were 95.1%, 1.1%, 1.4% and 3.6% for Enterobacterales, 91.2%, 4%, 4.4% and 4.7% for non-fermentative GNB, 93.4%, 7%, 6.1% and 0.3% for staphylococci and 94.5%, 2.8%, 4.1% and 1.7% for enterococci, respectively (Supplementary Table S26).

AST results were then analyzed per antimicrobial agents (Table 2). For Enterobacterales, ME and VME rates were below 6% for all antibiotics with 88.2% (15/17) of the ME and 75% (3/4) of the VME observed with β-lactams. For non-fermentative GNB, a VME rate of 25% (2/8) was obtained for levofloxacin with *Pseudomonas aeruginosa* and ME rates of 22.2% (2/9), 20% (2/10) and 16.7% (1/6) were observed with cefepime, piperacillin–tazobactam and trimethoprim–sulfamethoxazole, respectively (Table 2). For enterococcal strains, a VME was observed with linezolid (1/1) and ME rates of 11.1% (2/18) with levofloxacin, 8% (2/25) with ampicillin, 3.7% (1/27) with gentamicin and 4.8% (1/21) with vancomycin were observed (Table 2). More errors were obtained for *Staphylococcus* species with 23 errors occurring with oxacillin (ME of 50% (16/32) and VME of 18.9% (7/37)) (Table 2). However, the cefoxitin screen performed for *Staphylococcus aureus* and *Staphylococcus lugdunensis* was correct for all isolates. A high ME rate of 17% (8/48) was also observed with levofloxacin.

The QMAC-dRAST performs an ESBL screen only for *Escherichia coli*, *Klebsiella oxytoca*, *Klebsiella pneumoniae* and *Proteus mirabilis*. For these species, 100% (21/21) of ESBL screens were positive and 5.8% (3/52) of false positives were detected among non-ESBL producing isolates. The false positive ESBL screens occurred with a *K. oxytoca* K1 strain, a *K. pneumoniae* hyperproducing SHV-1 and a wild type *P. mirabilis*. For the 12 carbapenemase-producing GNB, resistance to β-lactams was well detected and no VME was reported in comparison to the Phoenix™ M50 AST results. For the 29 tested methicillin-resistant Staphylococcal isolates, VME errors were observed with oxacillin for *Staphylococcus aureus* but the cefoxitin screen was always positive. All the methicillin-resistant coagulase-negative *Staphylococcus* (ConS) were correctly identified with MIC values of oxacillin interpreted as resistant by the QMAC-dRAST.

### 3.2. Time to AST Results

**Figure 1:**
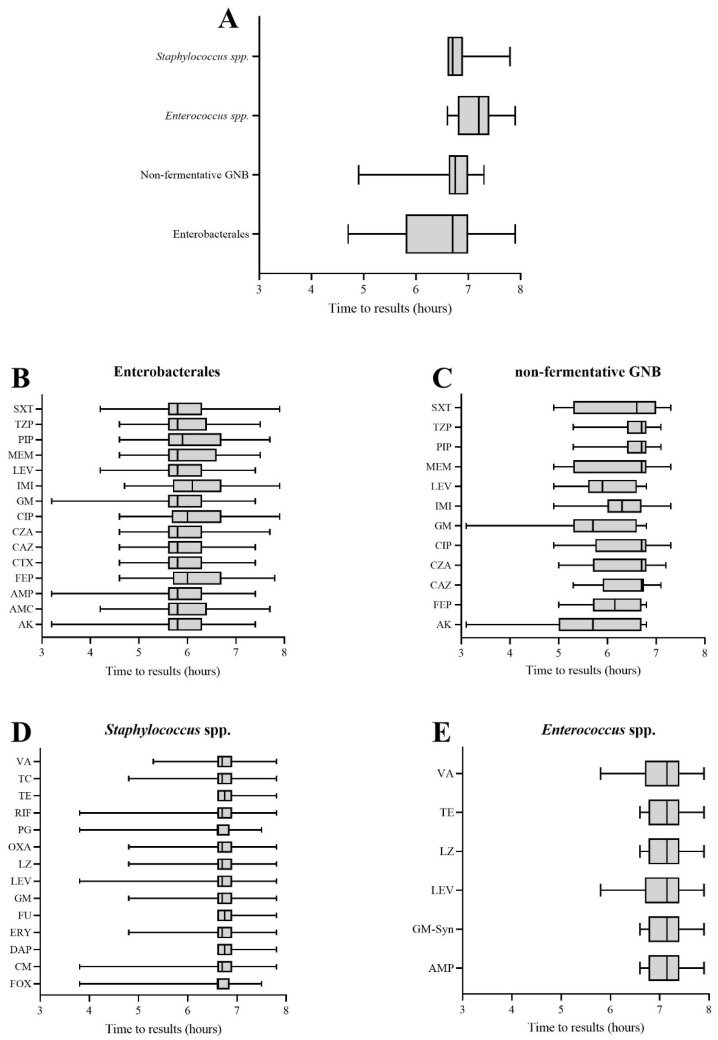
Median time to results for bacterial groups and antimicrobial agents. The box extends from the 25th to 75th percentiles and the whiskers from the smallest to the largest values. (**A**) Median time to results for the different bacterial classes. Median time to results per antibiotic for (**B**) Enterobacterales, (**C**) non-fermentative GNB, (**D**) *Staphylococcus* spp. and (**E**) *Enterococcus* spp. AK: amikacin, AMC: amoxicillin–clavulanate, AMP: ampicillin, CAZ: ceftazidime, CIP: ciprofloxacin, CM: clindamycin, CTX: cefotaxime, CZA: ceftazidime–avibactam, DAP: daptomycin, Ery: erythromycin, FEP: cefepime, FU: fusidic acid, GM: gentamicin, IMI: imipenem, LEV: levofloxacin, LZ: linezolid, MEM: meropenem, OXA: oxacillin, PG: penicillin G, PIP: piperacillin, RIF: rifampicin, TE: teicoplanin, TZP: piperacillin–tazobactam, SXT: trimethoprim–sulfamethoxazole, VA: vancomycin.

Median time to the AST result for the bacterial classes and antimicrobial agents is shown in Figure 1. The overall median turnaround time (TAT) was 6.7 h (range: 4.7–7.9). The median TAT was 6.7 h for Enterobacterales (range: 4.7–7.9), 6.7 h for non-fermentative GNB (range: 4.9–7.3), 6.7 h for staphylococci (range: 6.6–7.8) and 7.2 h for enterococci (range: 6.6–7.9) (Figure 1A). For GPC, results were more dispersed than for GNB. No significant difference in median TAT was observed between the different antibiotics tested for each bacterial group (Figure 1B–E).

### 3.3. Repeatability and Reproducibility

The results from the repeatability and reproducibility experiments are shown in Table 3 and Table 4. Sixteen strains were tested five times on the QMAC-dRAST. Overall, repeatability provided robust results with best and worst ratios of 98.8% and 99.6%, respectively, and mostly non-significant changes in MIC value interpretation. For GNB, 5.6% of MIC variations were within ±1 two-fold dilutions of the mode value and 0.3% within ±2 two-fold dilutions. For GPC, 7.7%, 1.4% and 0.9% of MIC variations were in a range of ±1, 2 and >2 two-fold dilutions, respectively, compared to the mode values. Reproducibility best and worst ratios were 95.0% and 98.3%, respectively. For GNB, 12%, 2.5% and 1.2% of MIC variations were within ±1, 2 or >2 two-fold dilutions of the mode value, respectively. For GPC, 17.5%, 4.1% and 2.5% of MIC variations were in a range of ±1, 2 and >2 two-fold dilutions, respectively, in comparison to the mode values.

## 4. Discussion

### 4.1. Main Findings

Compared to the BD Phoenix M50 AST and MIC gradient strips, the overall QMAC-dRAST CA for Enterobacterales, non-fermentative GNB, staphylococci and enterococci were 95.1%, 91.2%, 93.4% and 94.5%, respectively. The VME percentage was below 4% for all the groups except for staphylococci, which showed a VME rate of 7%. Compared to conventional AST, a significant decreased time to results was observed with a median of 6.7 h. Repeatability assays showed a high reliability of QMAC-dRAST AST results with best and worst ratios of 98.8% and 99.6%, respectively.

Most of the ME and VME observed with *Staphylococcus* species were due to oxacillin. The oxacillin MIC test and cefoxitin screen are essential for the detection of methicillin-resistant *Staphylococcus* strains. QMAC-dRAST AST includes both tests for *S. aureus* and *S. lugdunensis*, and for these species, all the methicillin-resistant strains were cefoxitin screen-positive despite oxacillin-susceptible MIC values. For coagulase-negative *Staphylococcus* (ConS), all the methicillin-resistant strains were associated with oxacillin-resistant MIC values, but 50% of ME was obtained for methicillin-susceptible ConS demonstrating an excellent sensitivity but a low specificity of methicillin-resistant ConS detection. Levofloxacin showed high rates of ME or VME with *Staphylococcus* spp., *Enterococcus* spp. and non-fermentative GNB. Therefore, a systematic verification of levofloxacin by another method would be required before reporting the final results. A previous study evaluating the former version of the QMAC-dRAST with GPC bacteria reported ME and VME with glycopeptides that we did not observe in comparison to the BD Phoenix M50 [22]. Importantly, no discrepancies were observed for glycopeptide resistance with VRE in our study.

For Enterobacterales, most of the ME and VME were obtained with β-lactams. For non-fermentative GNB, VME were obtained for levofloxacin and ME with cefepime, piperacillin–tazobactam and trimethoprim–sulfamethoxazole. Similar performances were reported by Grohs et al. with the former version of the QMAC-dRAST for Enterobacterales, but they observed more ME with non-fermentative bacteria and β-lactams [23]. ESBL producers were correctly detected by the QMAC-dRAST. As observed in this study, ESBL screening interference was observed and expected with K1 and SHV-1 producing strains as these enzymes are associated with a resistance to third generation cephalosporins. The QMAC-dRAST prototype used in this study was not equipped with an expert system for the detection of carbapenemase-producing strains. However, 10 out of the 12 carbapenemase-positive strains would have been detected by using the EUCAST meropenem screening cut-off of 0.125 mg/L for carbapenemase-producing Enterobacterales. The two meropenem MIC values inferior to 0.12 were obtained with OXA-48, a carbapenemase known for its weak hydrolytic activity [24,25,26].

Finally, repeatability experiments showed minor variations in MIC results, while reproducibility assays provided more dispersed MIC values, particularly with *Pseudomonas aeruginosa* and *Staphylococcus* spp. 

### 4.2. Limitation

Among the 250 ASTs performed with the QMAC-dRAST, 194 were performed from blood cultures spiked with well-characterized clinical isolates to increase the diversity of bacterial species and resistance phenotypes. Spiking of blood culture was standardized for the inoculum, incubation time and time delay processing of the sample upon blood culture positivity. Another extensive study should be conducted to provide a more precise performance of the QMAC-dRAST in the routine using patients’ positive blood culture so as to cover the diversity of pre-analytical and analytical parameters (clinical isolates, antimicrobial resistance phenotypes, infectious origin of the bacteremia, drug treatment, blood culture incubation time, time delay of positive blood culture processing) that may influence the performance of the QMAC-dRAST. In addition, the isolates tested in this study were characterized by a low prevalence of antibiotic resistance that may introduce a bias in the rate of ME and VME results.

### 4.3. Implication

The QMAC-dRAST system delivered AST results with an overall median time of 6.7 h (range: 4.7–7.9) allowing a rapid turnaround time for antibiotic regimen guidance. Significant benefits to the patients and hospitals with rapid AST systems are expected but would need to be analyzed in clinical studies, especially because these tests are more expensive than conventional AST methods. In countries with a low prevalence of antimicrobial resistance, the need for rapid AST can be debated as the maintenance of initial empirical antimicrobial therapy followed by antibiotic de-escalation are the strategies most applied for patients with severe sepsis that do not absolutely require rapid AST [27,28,29]. Studies considering patient clinical outcomes, the prevalence of antimicrobial resistance, organization of hospital units, therapeutic strategies and the cost of AST methods would be of interest to evaluate the benefits from rapid AST in healthcare systems.

## 5. Conclusions

In conclusion, the QMAC-dRAST is an easy-to-use system providing reliable AST performance. High performances were obtained with Enterobacterales and some improvements could be required for GPC, especially with ConS and oxacillin.

## Figures and Tables

**Table 1:** Distribution of bacterial species tested in the study and their associated antibiotic resistance mechanisms (*n* = 250).

Bacterial Species	N° Bacteria		Resistance Mechanisms										
	Spiking												
	No	Yes											
All	56	194	Wild Type	AmpC	ESBL	AmpC ESBL	AmpC Carbapenemase	Probable K1	Probable SHV-1	Carbapenemase	VRE	Methicillin-R	Total
**Enterobacterales, total**	28	102											130
*Escherichia coli*	16	15	17	2	12								31
*Klebsiella pneumoniae*	5	16	8		6				3	4			21
*Enterobacter cloacae*	0	14		7		4	3						14
*Proteus mirabilis*	2	9	10			1							11
*Klebsiella aerogenes*	1	9		10									10
*Klebsiella oxytoca*	1	9	5		2			3					10
*Serratia marcescens*	0	10		10									10
*Proteus vulgaris*	0	6		6									6
*Proteus hauseri*	0	5	5										5
*Citrobacter freundii*	1	3		3			1						4
*Morganella morganii*	1	3		4									4
*Citrobacter koseri*	0	3	3										3
*Salmonella* spp.	1	0	1										1
**Non-fermentative GNB, total**	5	15											20
*Pseudomonas aeruginosa*	4	9	11							2			13
*Acinetobacter baumannii*	0	5	3							2			5
*Acinetobacter* spp.	1	0	1										1
*Stenotrophomonas maltophilia*	0	1	1										1
***Staphylococcus* spp., total**	20	49											69
*Staphylococcus aureus*	9	15	9									15	24
*Staphylococcus epidermidis*	8	11	10									9	19
*Staphylococcus hominis*	2	7	7									2	9
*Staphylococcus capitis*	1	6	6									1	7
*Staphylococcus haemolyticus*	0	5	4									1	5
*Staphylococcus lugdunensis*	0	5	4									1	5
***Enterococcus* spp., total**	3	28											31
*Enterococcus faecium*	0	14	8								6		14
*Enterococcus faecalis*	3	10	12								1		13
*Enterococcus casseliflavus*	0	3									3		3
*Enterococcus gallinarum*	0	1									1		1
Total			125	42	20	5	4	3	3	8	11	29	250

N°: number, ESBL: extended spectrum beta-Lactamase, GNB: Gram-negative bacilli, K1: *Klebsiella oxytoca* isolates hyperproducing K1 β-lactamase, SHV-1: *Klebsiella pneumoniae* isolates hyperproducing SHV-1 β-lactamase, VRE: vancomycin-resistant *Enterococcus*.

**Table 2:** Performance characteristics of the QMAC-dRAST by antibiotic and bacterial group.

	N° of Antibiotics Tested	CA	CA%	me	me%	ME	ME%S	VME	VME%R	S	S_%	R	R_%
**Enterobacterales**													
Amikacin	130	130	100							130	100	0	0.0
Amoxicillin–Clavulanate	130	125	96.2			3	5.9	2	2.5	51	39.2	79	60.8
Ampicillin	130	130	100							16	12.3	114	87.7
Ceftazidime	130	114	87.7	11	8.5	5	5.1			98	75.4	32	24.6
Ceftazidime–Avibactam	129	129	100							129	100	0	0.0
Ciprofloxacin	129	123	95.3	5	3.9	1	1			104	80.6	25	19.4
Cefepime	130	116	89.2	9	6.9	5	4.6			108	83.1	22	16.9
Gentamicin	130	125	96.2	4	3.1			1	5.6	112	86.2	18	13.8
Imipenem	118	103	87.3	15	12.7					115	97.5	3	2.5
Levofloxacin	130	120	92.3	10	7.7					114	87.7	16	12.3
Meropenem	130	129	99.2			1	0.8			129	99.2	1	0.8
Piperacillin–Tazobactam	130	122	93.8	6	4.6	1	1	1	3.4	101	77.7	29	22.3
Trimethoprim–Sulfamethoxazole	130	128	98.5	1	0.8	1	1.1			89	68.5	41	31.5
**Non-fermentative GNB**													
Amikacin	19	18	94.7	1	5.3					16	84.2	3	15.8
Ceftazidime	13	13	100							8	61.5	5	38.5
Ceftazidime–Avibactam	12	12	100							9	75	3	25.0
Ciprofloxacin	19	17	89.5	2	10.5					14	73.7	5	26.3
Cefepime	13	11	84.6			2	22.2			9	69.2	4	30.8
Gentamicin	18	18	100							12	66.7	6	33.3
Imipenem	19	18	94.7	1	5.3					12	63.2	7	36.8
Levofloxacin	19	17	89.5					2	25	11	57.9	8	42.1
Meropenem	19	17	89.5	2	10.5					14	73.7	5	26.3
Piperacillin–Tazobactam	13	11	84.6			2	20			10	76.9	3	23.1
Trimethoprim–Sulfamethoxazole	7	4	57.1	2	28.6	1	16.7			6	85.7	1	14.3
***Staphylococcus* spp.**													
Clindamycin	69	67	97.1	1	1.4	1	1.8			58	84.1	11	15.9
Daptomycin	69	68	98.6			1	1.4			69	100	0	0.0
Gentamicin	69	67	97.1			1	2	1	5.6	51	73.9	18	26.1
Linezolid	69	69	100							69	100	0	0.0
Levofloxacin	69	60	87	1	1.4	8	17			48	69.6	21	30.4
Oxacillin	69	46	66.7			16	50	7	18.9	32	46.4	37	53.6
Penicillin G	24	24	100							1	4.2	23	95.8
Teicoplanin	69	68	98.6			1	1.5			65	94.2	4	5.8
Vancomycin	69	69	100							69	100	0	0.0
***Enterococcus* spp.**													
Ampicillin	31	26	83.9	3	9.7	2	8			25	80.6	6	19.4
Gentamicin-Syn	31	30	96.8			1	3.7			27	87.1	4	12.9
Linezolid	31	30	96.8					1	100	30	96.8	1	3.2
Levofloxacin	26	24	92.3			2	11.1			18	69.2	8	30.8
Teicoplanin	31	31	100							24	77.4	7	22.6
Vancomycin	31	30	96.8			1	4.8			21	67.7	10	32.3
Total	2604	2459	94.4	74	2.8	56	2.8	15	2.6	2025	77.77	579	22.2

GNB: Gram-negative bacteria, N°: number, me: minor error, ME: major error, ME%S: ME rate (percentage of major error divided by the number of susceptible strains), VME: very major error, VME%R: VME rate (percentage of very major error divided by the number of resistant strains), S: susceptible, R: resistant, S_%: percentage of antibiotics that exhibit cmi values interpreted as S or I. R_%: percentage of antibiotics that exhibit cmi values interpreted as R.

**Table 3:** Repeatability results obtained with the QMAC-dRAST.

		QMAC-dRAST MICs that Differed from the Mode Value by the Indicated Dilution						
		<−2	−2	−1	0	1	2	>2
**Enterobacterales**	**n**	0	0	11	454	15	0	0
	**%**	0	0	3.56	92.40	2.88	0.48	0
***Pseudomonas aeruginosa***	**n**	0	2	7	101	0	0	0
	**%**	0	1.82	6.36	91.82	0	0	0
***Staphylococcus* spp.**	**n**	0	1	14	336	10	5	4
	**%**	0	0.27	3.78	90.81	2.70	1.35	1.08
***Enterococcus* spp.**	**n**	0	0	5	60	5	0	0
	**%**	0	0	7.14	85.71	7.14	0	0
**Total**	**n**	0	3	37	961	30	5	4
	**%**	0	0.29	3.56	92.40	2.88	0.48	0.38

**Table 4:** Reproducibility results obtained with the QMAC-dRAST.

		QM-dRAST MICs that Differed from the Mode Value by the Indicated Dilution						
		<−2	−2	−1	0	1	2	>2
**Enterobacterales**	**n**	0	4	21	424	25	1	5
	**%**	0	0.83	4.38	88.33	5.21	0.21	1.04
***P. aeruginosa***	**n**	0	6	12	71	15	4	2
	**%**	0	5.45	10.91	64.55	13.64	3.64	1.82
***Staphylococcus* spp.**	**n**	0	10	40	277	27	5	11
	**%**	0	2.70	10.81	74.86	7.30	1.35	2.97
***Enterococcus* spp.**	**n**	0	3	6	57	4	0	0
	**%**	0	4.29	8.57	81.43	5.71	0	0
**Total**	**n**	0	23	79	829	71	10	18
	**%**	0	2.23	7.67	80.49	6.89	0.97	1.75

## Data Availability

Data supporting reported results will be available upon request for the peer-review process.

## Supplementary Materials

The following supporting information can be downloaded at: https://www.mdpi.com/article/10.3390/microorganisms10061212/s1, Table S1: List of antibiotics tested for each bacterial group included in the study; Table S2: Distribution of amikacin MICs obtained with the QMAC-dRAST versus the reference methods for gram-negative bacteria; Table S3: Distribution of ampicillin MICs obtained with the QMAC-dRAST versus the reference methods for gram-negative bacteria; Table S4: Distribution of amoxicillin-clavulanate MICs obtained with the QMAC-dRAST versus the reference methods for gram-negative bacteria; Table S5: Distribution of cefepime MICs obtained with the QMAC-dRAST versus the reference methods for gram-negative bacteria; Table S6: Distribution of ceftazidime MICs obtained with the QMAC-dRAST versus the reference methods for gram-negative bacteria; Table S7: Distribution of ceftazidime-avibactam MICs obtained with the QMAC-dRAST versus the reference methods for gram-negative bacteria; Table S8: Distribution of ciprofloxacin MICs obtained with the QMAC-dRAST versus the reference methods for gram-negative bacteria; Table S9: Distribution of gentamicin MICs obtained with the QMAC-dRAST versus the reference methods for gram-negative bacteria; Table S10: Distribution of imipenem MICs obtained with the QMAC-dRAST versus the reference methods for gram-negative bacteria; Table S11: Distribution of levofloxacin MICs obtained with the QMAC-dRAST versus the reference methods for gram-negative bacteria; Table S12: Distribution of meropenem MICs obtained with the QMAC-dRAST versus the reference methods for gram-negative bacteria; Table S13: Distribution of piperacillin-tazobactam MICs obtained with the QMAC-dRAST versus the reference methods for gram-negative bacteria; Table S14: Distribution of ampicillin MICs obtained with the QMAC-dRAST versus the reference methods for gram-positive bacteria; Table S15: Distribution of clindamycin MICs obtained with the QMAC-dRAST versus the reference methods for gram-positive bacteria; Table S16: Distribution of daptomycin MICs obtained with the QMAC-dRAST versus the reference methods for gram-positive bacteria; Table S17: Distribution of gentamicin MICs obtained with the QMAC-dRAST versus the reference methods for gram-positive bacteria; Table S18: Distribution of gentamicin-high MICs obtained with the QMAC-dRAST versus the reference methods for gram-positive bacteria; Table S19: Distribution of levofloxacin MICs obtained with the QMAC-dRAST versus the reference methods for gram-positive bacteria; Table S20: Distribution of linezolid MICs obtained with the QMAC-dRAST versus the reference methods for gram-positive bacteria; Table S21: Distribution of oxacillin MICs obtained with the QMAC-dRAST versus the reference methods for gram-positive bacteria; Table S22: Distribution of penicillin G MICs obtained with the QMAC-dRAST versus the reference methods for gram-positive bacteria; Table S23: Distribution of teicoplanin MICs obtained with the QMAC-dRAST versus the reference methods for gram-positive bacteria; Table S24: Distribution of tetracyclin MICs obtained with the QMAC-dRAST versus the reference methods for gram-positive bacteria; Table S25: Distribution of vancomycin MICs obtained with the QMAC-dRAST versus the reference methods for gram-positive bacteria; Table S26: Analysis of QMAC-dRast results for each bacterial species.

## Abbreviations

AST: antibiotic susceptibility testing, GPC: Gram-positive cocci, GNB: Gram-negative bacilli, CA: categorical agreement, VME: very major error, me: minor error, ME: major error, MIC: minimum inhibitory concentration, ESBL: extended spectrum β-lactamase, ConS: coagulase-negative *Staphylococcus,* S: susceptible, I: susceptible at increased exposure, R: resistant.
