# Supplementary material for: Performance Evaluation of the Quantamatrix QMAC-dRAST System for Rapid Antibiotic Susceptibility Testing Directly from Blood Cultures
Source: Microorganisms. 2022 Jun 14;10(6):1212. doi: 10.3390/microorganisms10061212 (PMC9229829; doi:10.3390/microorganisms10061212)
Supplement: Supplementary file 1 [file microorganisms-10-01212-s001.zip › microorganisms-1779733-supplementary.pdf]

Table S1: List of antibiotics tested for each bacterial group included in the study.

Enterobacterales	Non-fermentative GNB	*Staphylococcus spp.*	*Enterococcus spp.*
Amikacin	Amikacin	Clindamycin	Ampicillin
Amoxicillin-Clavulanate	Ceftazidime	Daptomycin	Gentamicin-Syn
Ampicillin	Ceftazidime-Avibactam	Gentamicin	Linezolid
Ceftazidime	Ciprofloxacin	Linezolid	Levofloxacin
Ceftazidime-Avibactam	Cefepime	Levofloxacin	Teicoplanin
Ciprofloxacin	Gentamicin[*]	Oxacillin	Vancomycin
Cefepime	Imipenem	Penicillin G	Erythromycin[**]
Gentamicin	Levofloxacin	Teicoplanin	Tetracyclin[**]
Imipenem	Meropenem	Vancomycin	Streptomycin high[**a]
Levofloxacin	Piperacillin -Tazobactam	Rifampicin[**]	
Meropenem	Trimethoprim-Sulfamethoxazole	Erythromycin[**]	
Piperacillin-Tazobactam	Piperacillin[**]	Tetracyclin[**]	
Trimethoprim-Sulfamethoxazole	Colistin[**]		
Piperacillin[**]	Cefotaxime[**a]		
Tetracyclin[**]			
Colistin[**]			
Cefotaxime[**a]			

GNB: Gram-negative bacteria.

[*] Antibiotic analyzed only in the QMAC-dRAST performance study. [**] Antibiotic analyzed only in the repeatability and reproducibility studies. [a] antibiotics not tested on the Phoenix™ M50.

Table S2: Distribution of amikacin MICs obtained with the QMAC-dRAST versus the reference methods for gram-negative bacteria.

		Reference MICs			
		**≤ 4**	**8**	**16**	**≥ 32**
**dRAST MICs**	**≤ 4**	136	10	0	0
	**8**	0	0	0	0
	**16**	0	0	0	0
	**≥ 32**	0	0	1	3

The reference method corresponds to the MICs measured with the Phoenix™ M50 or by gradient strip method.

Table S3: Distribution of ampicillin MICs obtained with the QMAC-dRAST versus the reference methods for gram-negative bacteria.

		Reference MICs			
		**≤ 2**	**4**	**8**	**≥ 16**
**dRAST MICs**	**≤ 4**	13	1	0	0
	**8**	1	1	0	0
	**≥ 16**	0	2	4	109

The reference method corresponds to the MICs measured with the Phoenix™ M50 or by gradient strip method.

Table S4: Distribution of amoxicillin-clavulanate MICs obtained with the QMAC-dRAST versus the reference methods for gram-negative bacteria.

		Reference MICs					
		**≤ 2**	**4**	**8**	**16**	**32**	**≥ 64**
**dRAST MICs**	**≤ 4**	26	10	2	0	0	0
	**8**	0	5	5	2	0	0
	**16**	0	0	1	8	0	1
	**≥ 32**	2	0	0	3	5	61

The reference method corresponds to the MICs measured with the Phoenix™ M50 or by gradient strip method.

Table S5: Distribution of cefepime MICs obtained with the QMAC-dRAST versus the reference methods for gram-negative bacteria.

		Reference MICs								
		**0.03125***	**0.0625**	**0.125**	**≤ 1***	**2**	**4**	**8**	**16**	**≥ 32**
**dRAST MICs**	**≤ 1**	0	0	0	88	0	0	0	0	0
	**2**	0	0	0	5	5	1	0	0	0
	**4**	0	0	0	2	1	4	1	0	0
	**8**	1	0	1	0	0	1	2	2	7
	**16**	0	0	0	0	0	0	1	1	3
	**≥ 32**	0	0	3	1	0	1	0	0	13

The reference method corresponds to the MICs measured with the Phoenix™ M50 or by gradient strip method. *Different lower MIC thresholds are provided by the Phoenix™ M50 according to the bacterial species.

Table S6: Distribution of ceftazidime MICs obtained with the QMAC-dRAST versus the reference methods for gram-negative bacteria.

		Reference MICs						
		**0.25***	**≤ 0.5***	**≤ 1***	**2**	**4**	**8**	**≥ 16**
**dRAST MICs**	**≤ 0.5**	1	65	1	0	0	0	0
	**1**	1	6	2	0	1	0	0
	**2**	0	0	5	4	2	1	1
	**4**	0	2	0	5	2	0	0
	**8**	0	1	0	0	3	1	2
	**16**	0	0	0	0	0	0	7
	**32**	1	1	0	0	0	1	9
	**≥ 64**	0	2	0	1	0	0	16

The reference method corresponds to the MICs measured with the Phoenix™ M50 or by gradient strip method. *Different lower MIC thresholds are provided by the Phoenix™ M50 according to the bacterial species.

Table S7: Distribution of ceftazidime-avibactam MICs obtained with the QMAC-dRAST versus the reference methods for gram-negative bacteria.

		Reference MICs								
		**≤ 0.0625**	**0.125**	**0.25**	**0.5**	**1**	**2**	**4**	**8**	**≥ 16**
**dRAST MICs**	**≤ 4**	2	0	81	28	14	9	0	0	0
	**8**	0	0	1	0	0	1	2	1	0
	**≥ 16**	0	0	0	0	0	0	0	0	3

The reference method corresponds to the MICs measured with the Phoenix™ M50 or by gradient strip method.

Table S8: Distribution of ciprofloxacin MICs obtained with the QMAC-dRAST versus the reference methods for gram-negative bacteria.

		Reference MICs							
		**0.015625***	**0.03125**	**≤ 0.0625***	**≤ 0.125***	**≤ 0.25***	**0.5**	**1**	**≥ 2**
**dRAST MICs**	≤ 0.0625	0	0	1	87	2	0	0	0
	0.125	0	0	0	9	2	0	0	0
	0.25	0	0	0	1	2	2	0	0
	0.5	0	0	0	0	1	7	0	0
	1	0	0	0	0	0	2	4	2
	2	0	0	0	0	0	0	4	5
	≥ 4	1	0	0	0	0	0	0	16

The reference method corresponds to the MICs measured with the Phoenix™ M50 or by gradient strip method. *Different lower MIC thresholds are provided by the Phoenix™ M50 according to the bacterial species.

Table S9: Distribution of gentamicin MICs obtained with the QMAC-dRAST versus the reference methods for gram-negative bacteria.

		Reference MICs			
		**≤ 1**	**2**	**4**	**≥ 8**
**dRAST MICs**	**≤ 1**	82	33	5	0
	**2**	0	1	1	1
	**4**	0	0	2	0
	**≥ 8**	0	0	0	24

The reference method corresponds to the MICs measured with the Phoenix™ M50 or by gradient strip method.

Table S10: Distribution of imipenem MICs obtained with the QMAC-dRAST versus the reference methods for gram-negative bacteria.

		Reference MICs						
		**≤ 0.25**	**0.5**	**1**	**2**	**4**	**8**	**≥ 16**
**dRAST MICs**	**≤ 0.125**	22	3	2	0	0	1	0
	**0.25**	23	8	3	0	1	0	0
	**0.5**	8	4	11	1	3	0	0
	**1**	1	2	6	7	0	0	0
	**2**	0	0	0	3	4	0	0
	**4**	0	0	5	0	4	0	0
	**8**	0	0	0	1	5	1	2
	**16**	0	0	0	0	0	0	1
	**≥ 32**	0	0	0	0	0	0	6

The reference method corresponds to the MICs measured with the Phoenix™ M50 or by gradient strip method.

Table S11: Distribution of levofloxacin MICs obtained with the QMAC-dRAST versus the reference methods for gram-negative bacteria.

		Reference MICs			
		**≤ 0.5**	**1**	**2**	**≥ 4**
**dRAST MICs**	**≤ 0.25**	102	2	0	0
	**0.5**	5	12	0	0
	**1**	1	4	3	0
	**2**	0	0	3	2
	**4**	0	0	0	2
	**≥ 8**	0	0	0	14

The reference method corresponds to the MICs measured with the Phoenix™ M50 or by gradient strip method.

Table S12: Distribution of meropenem MICs obtained with the QMAC-dRAST versus the reference methods for gram-negative bacteria.

		Reference MICs								
		**0.0625***	**≤ 0.125***	**0.25**	**≤ 0.5***	**1**	**2**	**4**	**8**	**≥ 16**
**dRAST MICs**	**≤ 0.125**	1	97	10	8	2	0	0	0	0
	**0.25**	0	1	2	3	1	1	0	0	0
	**0.5**	0	2	0	4	0	5	0	1	0
	**1**	0	1	0	0	0	1	0	0	0
	**2**	0	0	0	0	0	1	0	1	0
	**4**	0	0	0	0	0	0	0	1	0
	**8**	0	0	0	0	0	0	0	0	0
	**16**	0	0	0	0	0	0	0	0	0
	**≥ 32**	0	0	0	1	0	0	0	0	6

The reference method corresponds to the MICs measured with the Phoenix™ M50 or by gradient strip method. *Different lower MIC thresholds are provided by the Phoenix™ M50 according to the bacterial species.

Table S13: Distribution of piperacillin-tazobactam MICs obtained with the QMAC-dRAST versus the reference methods for gram-negative bacteria.

		Reference MICs								
		**1***	**2**	**≤ 4***	**8**	**16**	**≥ 32***	**64**	**≥ 128***	**≥ 256***
**dRAST MICs**	**≤ 8**	0	0	96	2	1	0	0	0	1
	**16**	0	0	0	3	5	1	0	0	0
	**32**	0	0	0	1	2	2	1	2	0
	**64**	0	0	0	0	0	1	1	2	0
	**≥ 128**	1	0	0	1	0	0	0	21	0

The reference method corresponds to the MICs measured with the Phoenix™ M50 or by gradient strip method. *Different lower or upper MIC thresholds are provided by the Phoenix™ M50 according to the bacterial species.

Table S14: Distribution of ampicillin MICs obtained with the QMAC-dRAST versus the reference methods for gram-positive bacteria.

		Reference MICs				
		0.5*	≤ 1.0*	≤ 2.0*	4	≥ 32
dRAST MICs	≤ 2	0	10	7	0	0
	4	0	3	0	0	0
	8	0	1	2	0	0
	≥ 16	1	0	0	1	6

The reference method corresponds to the MICs measured with the Phoenix™ M50 or by gradient strip method. *Different lower MIC thresholds are provided by the Phoenix™ M50 according to the bacterial species.

Table S15: Distribution of clindamycin MICs obtained with the QMAC-dRAST versus the reference methods for gram-positive bacteria.

		Reference MICs				
		0.125*	≤ 0.25*	0.5	1	≥ 2
dRAST MICs	≤ 0.125	1	55	1	0	0
	1	0	0	0	0	1
	≥ 2	0	1	0	0	10

The reference method corresponds to the MICs measured with the Phoenix™ M50 or by gradient strip method. *Different lower or upper MIC thresholds are provided by the Phoenix™ M50 according to the bacterial species.

Table S16: Distribution of daptomycin MICs obtained with the QMAC-dRAST versus the reference methods for gram-positive bacteria.

		Reference MICs		
		≤ 0.25	0.5	1
dRAST MICs	≤ 0.0625	1	0	0
	0.125	0	0	0
	0.25	9	5	0
	0.5	12	27	1
	1	1	10	2
	2	0	0	0
	4	0	0	0
	≥ 8	0	1	0

The reference method corresponds to the MICs measured with the Phoenix™ M50 or by gradient strip method.

Table S17: Distribution of gentamicin MICs obtained with the QMAC-dRAST versus the reference methods for gram-positive bacteria.

		Reference MICs					
		≤ 1.0	2	4	8	16	≥ 32
dRAST MICs	≤ 0.125	28	0	0	0	0	0
	0.25	17	0	0	0	0	0
	0.5	5	0	0	0	0	0
	1	0	0	1	0	0	0
	2	1	0	0	2	0	0
	≥ 4	0	0	0	14	0	1

The reference method corresponds to the MICs measured with the Phoenix™ M50 or by gradient strip method.

Table S18: Distribution of gentamicin-high MICs obtained with the QMAC-dRAST versus the reference methods for gram-positive bacteria.

		Reference MICs		
		64*	≤ 512*	≥ 1024
dRAST MICs	≤ 128	0	26	0
	≥ 256	1	0	4

The reference method corresponds to the MICs measured with the Phoenix™ M50 or by gradient strip method. *Different lower or upper MIC thresholds are provided by the Phoenix™ M50 according to the bacterial species.

Table S19: Distribution of levofloxacin MICs obtained with the QMAC-dRAST versus the reference methods for gram-positive bacteria.

		Reference MICs						
		0.125*	0.25	≤ 0.5*	1	2	4	≥ 8
dRAST MICs	≤ 0.125	0	0	10	0	0	0	0
	0.25	0	0	20	0	0	0	0
	0.5	0	0	9	1	0	0	0
	1	0	0	9	4	0	1	0
	2	0	0	2	1	2	1	0
	4	0	0	1	0	1	0	1
	8	0	0	1	0	1	2	6
	≥ 16	3	0	1	1	1	1	15

The reference method corresponds to the MICs measured with the Phoenix™ M50 or by gradient strip method. *Different lower or upper MIC thresholds are provided by the Phoenix™ M50 according to the bacterial species.

Table S20: Distribution of linezolid MICs obtained with the QMAC-dRAST versus the reference methods for gram-positive bacteria.

		Reference MICs			
		**1**	**2**	**4**	**≥ 8**
**dRAST MICs**	**≤ 0.5**	23	28	0	0
	1	24	19	5	1

The reference method corresponds to the MICs measured with the Phoenix™ M50 or by gradient strip method.

Table S21: Distribution of oxacillin MICs obtained with the QMAC-dRAST versus the reference methods for gram-positive bacteria.

		Reference MICs				
		**≤ 0.25**	**0.5**	**1**	**2**	**≥ 4**
**dRAST MICs**	**≤ 0.25**	1	0	0	0	0
	**0.5**	22	1	0	0	2
	**1**	2	3	2	3	4
	**2**	0	0	1	1	5
	**≥ 4**	0	0	1	1	20

The reference method corresponds to the MICs measured with the Phoenix™ M50 or by gradient strip method.

Table S22: Distribution of penicillin G MICs obtained with the QMAC-dRAST versus the reference methods for gram-positive bacteria.

		Reference MICs			
		**≤ 0.0625**	**0.01**	**0.25**	**≥ 0.5**
**dRAST MICs**	**0.125**	1	0	0	0
	**0.25**	0	0	1	0
	**≥ 1**	0	0	1	21

The reference method corresponds to the MICs measured with the Phoenix™ M50 or by gradient strip method.

Table S23: Distribution of teicoplanin MICs obtained with the QMAC-dRAST versus the reference methods for gram-positive bacteria.

		Reference MICs						
		≤ 0.5*	1	≤ 2*	4	8	≥ 16*	32*
dRAST MICs	≤ 0.125	1	0	0	0	0	0	0
	0.25	2	0	0	0	0	0	0
	0.5	40	0	0	0	0	0	0
	1	24	2	2	0	0	0	0
	2	1	0	10	2	0	0	0
	4	0	0	4	0	0	0	0
	8	0	0	0	1	0	3	0
	≥ 16	0	0	0	0	0	7	1

The reference method corresponds to the MICs measured with the Phoenix™ M50 or by gradient strip method. *Different lower or upper MIC thresholds are provided by the Phoenix™ M50 according to the bacterial species.

Table S24: Distribution of tetracyclin MICs obtained with the QMAC-dRAST versus the reference methods for gram-positive bacteria.

		Reference MICs			
		≤ 0.5	1	2	≥ 4
dRAST MICs	≤ 0.5	46	2	1	2
	1	0	1	4	2
	2	1	1	0	0
	4	0	0	0	0
	≥ 8	0	0	0	9

The reference method corresponds to the MICs measured with the Phoenix™ M50 or by gradient strip method.

Table S25: Distribution of vancomycin MICs obtained with the QMAC-dRAST versus the reference methods for gram-positive bacteria.

		Reference MICs										
		≤ 0.5	1	2	4	8	≥ 16*	32	64	128	256	≥ 512*
dRAST MICs	<=0.25	3	0	1	0	0	0	0	0	0	0	0
	0.5	12	16	0	1	0	0	0	0	0	0	0
	1	9	29	1	0	0	0	0	0	0	0	0
	2	0	4	11	1	0	0	0	0	0	0	0
	4	0	0	0	1	0	0	0	0	0	0	0
	8	0	1	0	0	1	1	0	0	0	0	0
	16	0	0	0	0	0	0	0	0	0	0	0
	>=32	0	0	0	0	0	7	0	0	0	0	1

The reference method corresponds to the MICs measured with the Phoenix™ M50 or by gradient strip method. *Different upper MIC thresholds are provided by the Phoenix™ M50 according to the bacterial species.

Table S26: Analysis of QMAC-dRast results for each bacterial species.

Bacterial species	N° of antibiotics tested	CA	CA %	me	me%	ME	ME% S	VME	VME %R	S_%
**Enterobacterales, total**	1676	1594	95.1	61	3.6	17	1.4	4	1.1	77.3
Escherichia coli	402	386	96	14	3.5	2	0.7			74.6
Klebsiella pneumoniae	272	263	96.7	8	2.9			1	1.2	68.8
Enterobacter cloacae	182	178	97.8	3	1.6			1	1.6	64.8
Proteus mirabilis	132	120	90.9	7	5.3	4	3.8	1	4.8	84.1
Klebsiella aerogenes	130	129	99.2	1	0.8					77.7
Klebsiella oxytoca	130	123	94.6	6	4.6	1	1			81.5
Serratia marcescens	130	115	88.5	9	6.9	6	5.6			83.1
Proteus spp.	142	132	93	7	4.9	2	1.7	1	8.3	91.5
Citrobacter freundii	52	51	98.1	1	1.9					86.5
Morganella morganii	52	48	92.3	2	3.8	2	5.4			78.8
Citrobacter koseri	39	36	92.3	3	7.7					92.3
Salmonella spp.	13	13	100							100
**Non-fermentative GNB, total**	171	156	91.2	8	4.7	5	4.4	2	4	70.8
Pseudomonas aeruginosa	128	119	93	3	2.3	4	4.4	2	5.7	72.7
Acinetobacter baumannii	35	30	85.7	4	11.4	1	5.6			62.9
Acinetobacter spp.	7	6	85.7	1	14.3					71.4
Stenotrophomonas maltophilia	1	1	100							100
***Staphylococcus*, total**	576	538	93.4	2	0.3	28	6.1	8	7.0	79.9
Staphylococcus aureus	216	205	94.9			4	2.4	7	14.6	77.8
Staphylococcus epidermidis	152	144	94.7			8	7.5			70.4
Staphylococcus hominis	72	65	90.3			6	9.4	1	12.5	88.9
Staphylococcus capitis	56	50	89.3			6	11.5			92.9
Staphylococcus haemolyticus	40	36	90	1	2.5	3	9.7			80
Staphylococcus lugdunensis	40	38	95	1	2.5	1	2.6			97.5
***Enterococcus*, total**	181	171	94.5	3	1.7	6	4.1	1	2. 8	80.1
Enterococcus faecium	83	75	90.4	3	3.6	4	7.4	1	3.4	65.1
Enterococcus faecalis	78	77	98.7			1	1.4			93.6
Enterococcus casseliflavus	15	14	93.3			1	7.1			93.3
Enterococcus gallinarum	5	5	100							80
**TOTAL**	2604	2459	94.4	74	2.8	56	2.9	15	2.6	77.7

GNB: gram negative bacteria, N°: number, CA: categorical agreement, me: minor error, ME: major error, ME%S: percentage of major error divided by the number of susceptible strains, VME: very major error, VME%R: percentage of very major error divided by the number of resistant strains, S_%: percentage of antibiotics which exhibit cmi values interpreted as S or I.
